# Comparison of the radiosensitivities of neurons and glial cells derived from the same rat brain

**DOI:** 10.3892/etm.2014.1802

**Published:** 2014-06-23

**Authors:** SHIGEHIRO KUDO, YOSHIYUKI SUZUKI, SHIN-EI NODA, TOSHIYUKI MIZUI, KATSUYUKI SHIRAI, MASAHIKO OKAMOTO, TAKUYA KAMINUMA, YUKARI YOSHIDA, TOMOAKI SHIRAO, TAKASHI NAKANO

**Affiliations:** 1 Department of Radiation Oncology, Gunma University Graduate School of Medicine, Maebashi, Gunma 371-8511, Japan; 2 Department of Neurobiology and Behavior, Gunma University Graduate School of Medicine, Maebashi, Gunma 371-8511, Japan; 3 Gunma University Heavy Ion Medical Center, Maebashi, Gunma 371-8511, Japan

**Keywords:** neuron, glia, radiation, apoptosis

## Abstract

Non-proliferating cells, such as mature neurons, are generally believed to be more resistant to X-rays than proliferating cells, such as glial and vascular endothelial cells. Therefore, the late adverse effects of radiotherapy on the brain have been attributed to the radiation-induced damage of glial and vascular endothelial cells. However, little is known about the radiosensitivities of neurons and glial cells due to difficulties in culturing these cells, particularly neurons, independently. In the present study, primary dissociated neurons and glial cultures were prepared separately from the hippocampi and cerebrum, respectively, which had been obtained from the same fetal rat on embryonic day 18. X-irradiations of 50 Gy were performed on the cultured neurons and glial cells at 7 and 21 days *in vitro* (DIV). The cells were fixed at 24 h after irradiation. Terminal deoxynucleotidyl transferase-mediated dUTP nick end labeling was then performed to measure the apoptotic indices (AIs). The AIs of non-irradiated and irradiated neurons at 7 DIV were 23.7±6.7 and 64.9±4.8%, and those at 21 DIV were 52.1±17.4 and 44.6±12.5%, respectively. The AIs of non-irradiated and irradiated glial cells at 7 DIV were 5.8±1.5 and 78.4±3.3% and those at 21 DIV were 9.6±2.6 and 86.3±4.9%, respectively. Glial cells and neurons were radiosensitive at 7 DIV. However, while glial cells were radiosensitive at 21 DIV, neurons were not.

## Introduction

Radiation therapy is among the essential treatment modalities for primary and metastatic brain tumors. However, subsequent cognitive function decline and developmental disorders following radiation therapy to the brain must be overcome, particularly in pediatric patients (1–5).

**Figure 1:**
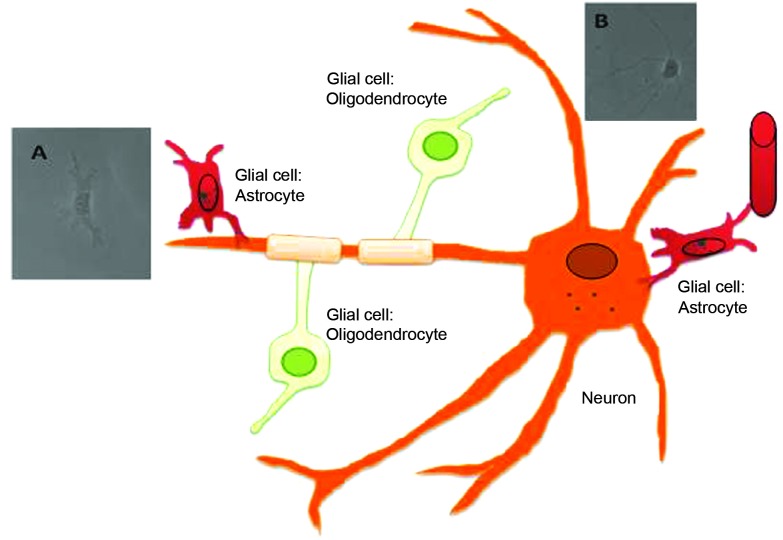
Illustration depicting the associations between neurons and glial cells in the CNS. The CNS is composed mainly of neurons and glial cells (astrocytes, oligodendrocytes and microglia). Glial cells are essential for the survival of neurons as they supply trophic factors to the neurons. (A and B) Phase contrast images show a (A) glial cell and (B) neuron. CNS, central nervous system. Magnification, ×20.

The central nervous system (CNS) is composed mainly of neurons, glial cells and vascular endothelial cells (Fig. 1). Neurons, the majority of which cease cell proliferation during fetal development, have been considered to be more radioresistant than glial and vascular endothelial cells, which continue to proliferate subsequent to birth. Molecular studies have provided evidence that glial cells are essential for the survival of neurons by supplying trophic factors to the neurons (6–9). Thus, the mechanism underlying the late adverse brain effects of radiation therapy has been believed to mainly be the insufficient supply of nutrients and blood to neurons due to the impaired functions of irradiated glial and vascular endothelial cells, rather than a direct effect of the radiation itself on neurons.

It was shown in the late 1990s that adult neurogenesis occurs in certain areas of the brain, including the subventricular zone (SVZ) and subgranular layer (SGL) (10). In addition, radiation-induced apoptosis of neural progenitor cells was observed in the SVZ and SGL, and relatively high radiosensitivity was demonstrated in neurons residing in the areas where neurogenesis occurs (11,12). These findings have raised the possibility that radiation-induced neuronal death may be one of the causes of the late adverse effects of radiation therapy, such as functional and developmental disorders. Therefore, attempts have been made to prevent adverse effects from developing following cranial irradiation using intensity-modulated radiation therapy to reduce the dose to areas that may be highly radiosensitive, such as the SVZ and SGL (13–16).

Radiation affects neurons and glial and vascular endothelial cells. It is therefore difficult to evaluate the radiation sensitivities of these cell types separately in *in vivo* studies. Furthermore, the majority of the previously reported *in vitro* studies were conducted on a mixture of neurons and glial cells (17). However, due to technical difficulties, only a few investigations, including our previous studies (18,19), have examined the radiosensitivity of neurons by employing monocultures of this cell type alone.

To estimate the extent of the involvement of neurons and glial cells in the adverse brain effects of radiation therapy, it is essential to compare radiosensitivities between glial cells and neurons. A previous study using glial cells and neurons cultured separately for such a comparison demonstrated the radiosensitivity of glial cells to be comparable to that of neurons (17). However, the cells used in that study were isolated from different individuals (different genetic backgrounds) and cultured for different lengths of time (different developmental stages), such that the results are not entirely convincing. In the present study, neurons and glial cells were therefore obtained from the same rat to ensure uniform conditions (identical genetic backgrounds and developmental stages) and their radiosensitivities were investigated separately.

## Materials and methods

### Cell culture

The modified Banker’s method was used for primary neuronal cultures (20). Briefly, cells were obtained from the hippocampi of Wistar rat fetuses (Imai Jikkendobutu Shiikujo, Saitama, Japan) at embryonic day 18, treated with trypsin and mechanically dispersed by trituration with Pasteur pipettes. The cells were then seeded at a density of 5,000 cells/cm^2^ on glass coverslips coated with poly-L-lysine and cultured in minimum essential medium (MEM; Invitrogen Life Technologies, San Diego, CA, USA) for 3 h. The coverslips were then transferred to culture dishes containing a monolayer of supporting glial cells maintained in serum-free MEM supplemented with B27 (Invitrogen Life Technologies). Cytosine β-D-arabinofuranoside (Sigma, St. Louis, MO, USA) (10 μM) was added to the culture medium at 3 days *in vitro* (DIV) to inhibit glial cell proliferation. Neurons were irradiated at 7 or 21 DIV. For X-irradiation of the cells, the cover slips were transferred to another culture dish containing only medium and no glial cells. Immediately subsequent to the irradiation of the neurons, the cover slips were returned to the original culture dishes. Although the neurons were in direct contact with the glial cells, they were easily separable; thus, it was possible to irradiate and observe only neurons.

Glial cells were obtained from the cerebral cortex of the same Wistar rat fetus as that used for obtaining neurons at embryonic day 18. Briefly, the cells were treated with trypsin, dispersed by trituration with Pasteur pipettes and then seeded at a density of 5,000 cell/cm^2^ on glass coverslips coated with poly-L-lysine and cultured in MEM. Four days later, the cells were again treated with trypsin, dispersed with Pasteur pipettes and then cultured in new MEM. Glial cells were also irradiated at 7 DIV or 21 DIV. For X-irradiation of the cells, the cover slips were transferred to another culture dish containing only medium and no glial cells. Following irradiation of the glial cells, the cover slips were returned to the original culture dishes.

All animal experiments were performed in accordance with the guidelines set by the Animal Care and Experimentation Committee (Gunma University, Maebashi, Japan).

### Irradiation and cell fixation

At 7 and 21 DIV, neural and glial cells were irradiated with 200 kV X-rays (Siemens-Asahi Medical Technologies Ltd., Tokyo, Japan) at a dose of 50 Gy. At 24 h after irradiation, the cells were fixed in 4% paraformaldehyde for 24 h at 4°C. Non-irradiated culture cells were handled in parallel with the irradiated samples as a control.

### Assessment of apoptosis

Apoptosis was determined by terminal deoxynucleotidyl transferase-mediated dUTP nick end labeling (TUNEL) assay using the ApopTag^®^ Plus *In Situ* Apoptosis Fluorescein Detection kit (Chemicon International, Temecula, CA, USA). Fixed cells on coverslips were permeabilized in ethanol:acetic acid (2:1) for 15 min at −20°C. The cells were then washed twice with phosphate-buffered saline (pH 7.4) for 5 min and incubated with ApopTag equilibration buffer for 5 min, followed by terminal deoxynucleotidyl transferase linkage of digoxigenin-tagged dUTP to the 3′-OH termini of DNA fragments at 37°C for 60 min. The reaction was terminated at 37°C in stop/wash buffer for 30 min and the cells were then washed. Subsequent to washing, the cells were incubated with anti-digoxigenin fluorescein antibody for 30 min and the coverslips were then mounted on slides with Vectashield^®^ Mounting Medium with DAPI (Vector Laboratories, Burlingame, CA, USA).

### Evaluation method

Fluorescein-labeled cells were observed under a Zeiss Axioplan microscope (Carl Zeiss AG, Jena, Germany) equipped with a Photometrics CoolSnap FX cooled CCD camera (Photometrics, Tucson, AZ, USA) using MetaMorph software (Universal Imaging Corp., West Chester, PA, USA). Apoptotic cells were counted on each slide. The apoptotic index (AI) was calculated as the number of DAPI- and TUNEL-positive cells divided by the number of DAPI-positive cells. The cells positive for TUNEL and negative for DAPI were excluded from the calculations.

### Statistical analysis

Statistical analysis was performed using StatMate software (GraphPad Software, Inc., San Diego, CA, USA). P<0.01, as determined using a Student’s t-test, was considered to indicate a statistically significant difference. All results are shown as the mean ± standard deviation.

## Results

**Figure 2:**
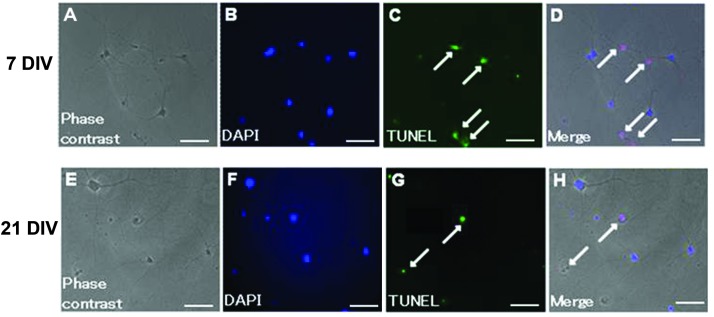
TUNEL analysis of cells undergoing radiation-induced apoptosis. (A–D) 7 DIV neurons; (E–H) 21 DIV neurons. (A and E) Phase contrast images; (B and F) DAPI fluorescence images; (C and G) TUNEL fluorescence images; (D and H) double fluorescence images for TUNEL (red) and DAPI (blue). Scale bar = 50 μm. DIV, days *in vitro*; TUNEL, terminal deoxynucleotidyl transferase-mediated dUTP nick end labeling. Magnification, ×20. The arrows indicate nuclear pylnosis.

**Figure 3:**
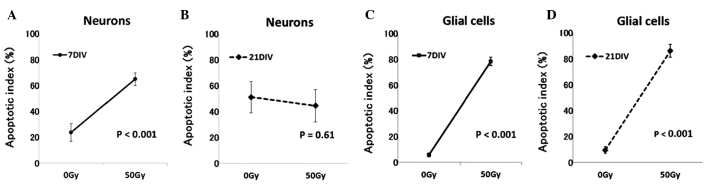
Plots of the average AIs of cells irradiated with 0 Gy (Control) or 50 Gy, at 7 or 21 DIV. Neurons: (A) The AI was significantly increased by 50 Gy irradiation at 7 DIV as compared with the control (n=3, P<0.001). (B) No increase in AI was identified at 21 DIV (n=8, P=0.61). Glial cells: The AI increased significantly with 50 Gy irradiation at both (C) 7 DIV and (D) 21 DIV (7 DIV, n=3, P<0.001; 21 DIV, n=4, P<0.001). The bars represent standard deviations. DIV, days *in vitro*; AI, apoptotic index.

The average numbers of neurons and glial cells counted in each coverslip were 332 (range, 211–556) and 273 (range, 131–292), respectively. Representative images of irradiated neurons are shown in Fig. 2. The AI of 7 DIV neurons was 23.7±6.7% (n=3) in the control group and significantly higher, 64.9±4.8% (n=3), in the 50 Gy irradiated group (P<0.001) (Fig. 3A). At 21 DIV, the AI of neurons was 52.1±17.4% (n=9) in the control group and 44.6±12.5% (n=8) in the irradiated group; no significant difference was identified in the number of apoptotic cells between the two groups (P=0.61) (Fig. 3B). The average AI of 7 DIV glial cells was 5.8±1.5% (n=3) in the control group and 78.4±3.3% (n=3) in the 50 Gy irradiated group (Fig. 3C), and the average AI of 21 DIV glial cells was 9.6±2.6% (n=4) in the control group and 86.3±4.9% (n=4) in the 50 Gy irradiated group (Fig. 3D). The differences between the control and 50 Gy irradiated groups were significant at 7 DIV (P<0.001) as well as at 21 DIV (P<0.001).

Comparisons at the corresponding time-points revealed both glial cells and neurons to be radiosensitive at 7 DIV, whereas glial cells but not neurons were radiosensitive at 21 DIV.

## Discussion

Our previous study revealed 7 DIV neurons (morphologically and functionally immature cells) to be relatively radiosensitive, while 21 DIV neurons (morphologically and functionally mature cells) were found to be extremely radioresistant, showing no increase in apoptosis even following high-dose irradiation (19). Furthermore, when 7 DIV neurons were exposed to low doses of X-irradiation (0, 5, 4 and 10 Gy) and further cultured for 14 and 21 days in total, the number of apoptotic cells increased, and the clustering of synaptic proteins, indicative of the maturation of synapses, decreased dose-dependently following irradiation (18). Consistent with our previous findings, the present results showed that the number of 7 DIV neurons undergoing apoptosis increased following irradiation, whereas radiation did not significantly increase apoptosis in 21 DIV neurons. These results indicate that radiosensitive immature neurons become radioresistant with maturation and that mature neurons are radioresistant.

The AI of glial cells did not differ significantly between 7 and 21 DIV in this study. Although no study has focused on the association between the maturity and radiosensitivity of glial cells, if the maturities of these cells reflect their radiosensitivity, our present results may suggest their maturities to be similar at 7 and 21 DIV. In other words, since glial cells have the ability to proliferate (gliogenesis), unlike neurons, it is assumed that a glial cell population would represent a mixture of cells with differing maturities due to this proliferation. Thus, their similar radiosensitivities suggest that the glial cells in this study may have been at similar maturation stages.

Following irradiation, glial cells may undergo mitotic cell death. Furthermore, we observed in a previous study that a large percentage of neurons underwent delayed apoptosis subsequent to irradiation (18). Thus, comparing the radiosensitivities of neurons and glial cells based on their AIs at 24 h after irradiation can be difficult. A number of studies have shown that the ability to repair radiation damage differs among sites (10,21). Eriksson *et al* (10) reported that adult neurogenesis occurs in both the SVZ and the SGL, and Seaberg *et al* (21) showed that stem cells with pluripotency and self-renewal ability were present in the SVZ, while the SGL contained predominantly neural progenitor cells without pluripotency and fewer stem cells. In other words, due to the presence of radioresistant and pluripotent stem cells in the SVZ, neurogenesis may occur following irradiation in this area, whereas recovery subsequent to irradiation may be poor in the SGL where the number of the stem cells is limited. In a study by Hellström *et al* (22), the volume and rate of DNA synthesis following whole brain irradiation were reported to be significantly higher in the SVZ than in the SGL (22). Glial cells have the ability to proliferate, such that damaged glial cells can be replaced by gliogenesis. Therefore, to understand the mechanisms underlying the adverse effects of radiation therapy on the brain, the neurogenesis, restoration of glial cells and secondary effects due to brain blood vessels being impaired by irradiation must all be taken into consideration. Furthermore, radiation effects on the brain may vary according to the irradiation site, extent and dose, due to the heterogeneous distribution of neural stem cells.

In conclusion, the radiosensitivities of neurons and glial cells, obtained from the same rat brain, were evaluated by examining the number of cells undergoing radiation-induced apoptosis. The results showed both glial cells and neurons to be radiosensitive at 7 DIV, whereas only glial cells were radiosensitive at 21 DIV; neurons exhibited radioresistance at 21 DIV. Further studies are required to elucidate the mechanisms underlying the late adverse effects of radiation therapy on the CNS.
